# An unusual and difficult diagnosis of intestinal obstruction: The abdominal cocoon. Case report and review of the literature

**DOI:** 10.1186/1749-7922-1-8

**Published:** 2006-03-24

**Authors:** Ali O Devay, Ismail Gomceli, Birol Korukluoglu, Ahmet Kusdemir

**Affiliations:** 1 Department of general surgery, 2^nd ^General surgery clinic, Ankara Ataturk training and research hospital, Ankara, Turkey; 2 Department of general surgery, 1^st ^General surgery clinic, Ankara Ataturk training and research hospital, Ankara, Turkey; 3 Isci Bloklari Mah. 381. Sok. Idareciler Sitesi, C Blok No.17, Karakusunlar 06520, Ankara, Turkey

## Abstract

Abdominal cocoon is a rare cause of intestinal obstruction. The abdominal cocoon is probably a developmental abnormality, largely asymptomatic, and is found incidentally at laparotomy or autopsy. Pre-operative diagnosis cannot be often made correctly. This rare entity of intestinal obstruction has been described in the whole literature as a thick fibrotic sac covering the small bowel partially or completely. The etiology of abdominal cocoon is unknown and most often it is found in adolescent girls from tropical or subtropical countries. Complete recovery is generally expected after the removal of the membrane surgically. This paper reports a male patient who has had intestinal obstruction symptoms and has per-operatively been diagnosed as abdominal cocoon.

## Background

"Abdominal cocoon" is a rare condition of unknown etiology that primarily affects adolescent girls living in tropical and subtropical regions. The abdominal cocoon was first described and named in 1978 by Foo *et al *[1]. Since that report in 1978, which argued that the abdominal cocoon was caused by a low-grade peritonitis from retrograde menstruation, the etiology of this entity has remained relatively unknown. Later, there have been isolated case reports involving older patients and in both genders discussed in literature [2-5]. A Medline search revealed that in English literature approximately 47 cases have been reported up to now (See Additional file 1). In each of these cases, the small bowel becomes encased in a thick membrane. The accessory membrane, present in front of the small bowel, can be easily removed, but excision may not be necessary. Generally, complete recovery is the rule after the removal of the membrane. This paper reports one male patient who presented an emergency with features of intestinal obstruction.

## Case presentation

A 30-year-old male patient was admitted to the Emergency Department of our hospital with a 24-hour history of abdominal pain which had developed suddenly and initially been colicky in nature, with intermittent vomiting and abdominal distension. There was no history of peritonitis, abdominal surgery or tuberculosis. There had been two similar episodes in one year. But, because of the spontaneous regression of the symptoms, he was not examined by any physician in those episodes. Because of the asthma and hypertension, he had used calcium canal blockers and teophiline for two years.

General physical examination revealed an anxious looking and mild dehydration. His pulse was 110/minute, temperature 37.8°C, blood pressure 130/70 mmHg. There was no cyanosis or jaundice. No abnormalities of the chest or cardiovascular system were found. Local examination of the abdomen revealed a distended abdomen, hypoactive bowel sounds, mild tenderness and rigidity in the whole abdomen. A tender lump was palpated in the umbilical region which moved from side to side with mild guarding. There was no hepatomegaly or splenomegaly and rectal examination was unremarkable.

Routine laboratory workup revealed a total leukocyte count of 13300 cells/ml, hemoglobin of 17.9 g %, and normal serum chemistry and normal urine analysis. PA X-ray of the chest was normal but plain standing X-ray of the abdomen showed multiple air-fluid levels with no free gas under the diaphragm. An ultrasound examination of the abdomen showed normal liver, gallbladder, pancreas and kidneys. But there was a small amount of fluid in the pelvis and a heterogen, vascularized mass lesion was seen in the periumblical region which had 96 × 75 mm dimensions.

Under a provisional clinical diagnosis of mechanical small bowel obstruction, emergency laparotomy was performed through a midline incision. After opening the peritoneum, especially the proximal section of the small bowel was observed to be dilated, its mesentery was edematous and the whole small bowel was covered by a dense whitish and approximately 2 mm thick membrane which gave the appearance of a cocoon (Figure). This membrane was freely mobile freed from the parietal peritoneum. There was a straw-colored fluid around 200 ml in the pelvis. The membrane enveloping the small bowel was incised carefully and separated from the intestinal serosa by sharp and blunt dissections and the whole small bowel was freed and followed from treitz to ileocaecal junction. Partial omentectomy and appendectomy were performed. The whole freed small bowel was viable and the colon was normal; thus no further surgical procedure was deemed necessary. The peeled-off membrane was studied histopathologically and microscopic examination revealed a chronic non-specific inflammatory reaction and mature fibrous tissue.

The patient had an uneventful post-operative period and was discharged from the hospital on the seventh post-operative day. He has been regularly attending our follow-up clinics for the last one year and has remained free of bowel symptoms throughout this period.

## Conclusion

The etiologies of intestinal obstruction differ very little from one part of the globe to another. Adhesion formation causing intestinal obstruction is a very important and frequently observed pathology. The case reported by us in this paper is peculiar and is presented with unusual types of adhesions.

In 1978 Foo *et al*. described a curious condition by 10 cases of adolescent females with acute or subacute small bowel obstruction symptoms named as "abdominal cocoon" [1]. A Medline search revealed that so far approximately 47 cases have been reported in English literature (See Additional file 1). In each of these cases, the small bowel becomes encased in a thick membrane. The membrane present in front of the small bowel can be easily removed, but excision may not be necessary. In general, complete recovery is the rule after the removal of the membrane. The affected individuals are usually from tropical and subtropical regions [6]. The condition usually affects adolescent girls ranging in age from 6 to 18 years; however, there have been anecdotal reports of a higher age range. Regarding sex distribution, this condition is generally found exclusively in females, although cases in males have also been reported as in our case report. These patients usually present with features of acute or subacute small bowel obstruction, symptoms of chronic obstruction and weight loss, and/or pain associated with an abdominal lump. A pre-operative diagnosis is almost never made and the non-specific and intermittent symptoms may result in delay in diagnosis. Laboratory and radiological studies are non-specific, although plain radiographs of the abdomen may suggest features of intestinal obstruction [6]. Most cases are diagnosed when a laparotomy is performed for obstructive symptoms [7-9].

The characteristic finding is that of the encasement of the whole or part of the small bowel by a thick shiny membrane, aptly simulating a cocoon. The loops of the small bowel have been stuck together by filmy soft adhesions separated easily by blunt or sharp dissections from the cocoon [3].

Surgery remains the cornerstone in the management of abdominal cocoon. Careful dissection and excision of the thick sac with the release of the small intestine leads to complete recovery. Resection of the bowel is indicated only if it is nonviable [2]. An incidental appendectomy is recommended, as the appendix would be difficult to find should the patient develop acute appendicitis in the future [6,14]. We performed appendectomy in our case because of this reason.

To explain the etiology and the formation of the membrane of this condition a number of hypotheses have been proposed. These include retrograde menstruation with a superimposed viral infection [1], retrograde peritonitis via the fallopian tubes, and cell-mediated immunological tissue damage incited by gynecological infection [10]. However, none of these hypotheses explain the characteristic age group, sex, and geographical distribution of this disease and there is no objective evidence to substantiate them.

In literature some authors define these cases by different diagnoses such as 'encapsulating sclerosing peritonitis', 'abdominal cocoon', 'peritoneal encapsulation' or they use all these definitions for one patient. The abdominal cocoon should not be confused with "encapsulating sclerosing peritonitis", which occurs in association with the use of the beta blocker practolol, cirrhosis of the liver, chronic peritoneal dialysis, carcinoid syndrome, familial Mediterranean fever and asbestos exposure [6,11,12]. Although commonly reported in young girls, it is also seen in elderly male patients. In this entity the whole peritoneal cavity is often obliterated by dense adhesions. The visceral and parietal peritoneums are thickened with a shortened small bowel encased in a rigid tube. There is usually no plane of cleavage between the membrane and the bowel making the release of the bowel loops extremely difficult.

'Peritoneal encapsulation' is a related condition of abdominal cocoon but it differs in that small or large bowel is found behind an accessory, but otherwise normal, peritoneal membrane [13]. This condition has been reported in elderly males and as it is usually asymptomatic, it is generally discovered either at laparotomy for an unrelated condition or at autopsy. The peritoneal membrane in this entity is attached to the ascending and descending colon laterally, the transverse mesocolon cranially, and to the posterior parietal peritoneum caudally. The membrane has two openings, one as the intestine enters at about the duodenojejunal junction and the other as it leaves at the ileocecal junction. The relative position of the viscera is normal. Peritoneal encapsulation is probably an embryologic abnormality. The accessory peritoneal sac may derive from the peritoneum of the yolk-sac as it is withdrawn rather rapidly into the abdominal cavity with the small bowel behind it during the 12^th ^week of gestation [14,15].

The prognosis of abdominal cocoon after surgery seems excellent and no recurrence has been described. Only one patient in literature who had presented with long-standing symptoms and weight loss died post-operatively after subclavian vein thrombosis due to intravenous hyperalimentation [1].

**Figure 1:**
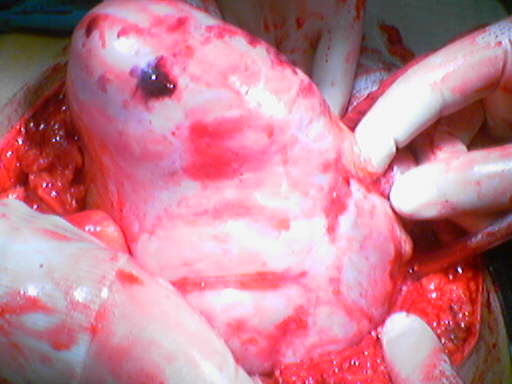
Intraoperative appearance of small bowel which was covered by a dense whitish membrane as a cocoon.

## Supplementary Material

Additional File 1 The table includes clinical summaries of the cases which were diagnosed as abdominal cocoon in English literature up to now. (*: No information about these data could be found by Medline research). Click here for file
