# An unusual and difficult diagnosis of intestinal obstruction: The abdominal cocoon. Case report and review of the literature

**DOI:** 10.1186/1749-7922-3-36

**Published:** 2008-12-17

**Authors:** Ali O Devay, Ismail Gomceli, Birol Korukluoglu, Ahmet Kusdemir

**Affiliations:** 1 Department of general surgery, 2nd General surgery clinic, Ankara Ataturk training and research hospital, Ankara, Turkey; 2 Department of general surgery, 1st General surgery clinic, Ankara Ataturk training and research hospital, Ankara, Turkey

## Abstract

Since publication of our article, "An unusual and difficult diagnosis of intestinal obstruction: The abdominal cocoon. Case report and review of the literature." World J Emerg Surg. 2006, 1: 8 we believe that the case mentioned should have been described as a 'peritoneal encapsulation' rather than 'abdominal cocoon' as concluded in the original publication.

## 

Since publication of our article [1] we believe that the case mentioned should have been described as a 'peritoneal encapsulation' rather than 'abdominal cocoon' as concluded in the original publication [1].

The abdominal cocoon is a condition in which a variable length of healthy small bowel is enveloped in a fibrocollagenous membrane, giving the appearance of a cocoon. It is an unusual cause of intestinal obstruction in children and adolescents. It was first described and named in 1978 by Foo et al. [2].

Peritoneal encapsulation was first described by Cleland in 1868 [3]. Peritoneal encapsulation is largely asymptomatic and found incidentally at laparotomy or autopsy as abdominal cocoon. The accessory membrane present in front of the small bowel. The small intestine lying behind an accessory but otherwise normal peritoneal membrane.

In our article [1] the pathology was described as the whole small bowel being covered by a dense whitish and approximately 2 mm thick membrane which gave the appearance of a cocoon and it was named as abdominal cocoon. However an accessory membrane was seen in clinical picture. But abdominal cocoon is a condition characterized by a total or partial encasement of the small bowel by a fibrocollagenous cocoon-like sac and it is necessary to free this fibrocollagenous tissue from the serosa of the intestine. The accessory membrane is related with the peritoneal encapsulation and there is no fibrocollagenous tissue on the serosa of intestine and incising this membrane is adequate for treatment. It is difficult to enter the abdominal cavity and there is no plane of cleavage between the membrane and the bowel because of this accessory membrane in peritoneal encapsulation. However there is a plane of cleavage in an abdominal cocoon and serosal fibrocollagenous tissue is a unique pathology of this condition.

There are other case reports that have confused these two entities by mistake. For example Sayfan et al. [4] described a case of peritoneal encapsulation giving rise to acute mechanical small bowel obstruction in a 12-year-old girl. But Sieck et al. [5] showed that the patient mentioned in this article had abdominal cocoon, a condition with an entirely separate anatomy, etiology, clinical picture and prognosis.
