# Supplementary material for: An unusual and difficult diagnosis of intestinal obstruction: The abdominal cocoon. Case report and review of the literature
Source: World J Emerg Surg. 2006 Mar 24;1:8. doi: 10.1186/1749-7922-1-8 (PMC1459267; doi:10.1186/1749-7922-1-8)
Supplement: Additional File 1 — The table includes clinical summaries of the cases which were diagnosed as abdominal cocoon in English literature up to now. (*: No information about these data could be found by Medline research). [file 1749-7922-1-8-S1.doc]

| **Reference** | **Nb.of**  **cases** | **Age**  **(years)** | **Sex**  **M F** | **Symptoms** | **Abdominal**  **observations** | **Operative**  **findings** | **Operative**  **procedure** | **Outcome** |
| --- | --- | --- | --- | --- | --- | --- | --- | --- |
| 1 | 10 | 13 - 18 | - 10 | Abdominal pain, vomiting, distension, constipation | Six patients with abdominal mass, no mass in four patients | Cocoon in distal part of small bowel in 6 patients, cocoon of whole of small bowel in 4 patients | Lysis in seven patients, Noble’s plication in one, ileotransverse colostomy in one, limited hemicolectomy in one | * |
| 16 | 1 | 4 | - 1 | Symptoms of obstruction | Abdominal mass | * | * | * |
| 17 | 1 | 17 | - 1 | Abdominal pain | Abdominal mass | Cocoon of distal ileum | Lysis | * |
| 14 | 1 | 14 | - 1 | Distension, vomiting | No mass | Cocoon of whole of small bowel | Lysis | Well on discharge |
| 18 | 1 | 16 | - 1 | Abdominal pain, vomiting constipation | Abdominal mass in left and right iliac fossa | Cocoon of whole of small bowel | Lysis | Well on discharge |
| 19 | 1 | 15 | - 1 | Periumblical pain, vomiting | Central abdominal mass | Cocoon of liver, stomach, whole ileum and distal jejunum | Lysis | Well on discharge |
| 3 | 5 | 13 - 43 | 1 4 | Abdominal pain, distension, vomiting | Three patients with abdominal mass | Cocoon of whole of small bowel | Lysis in all patients | Well on discharge |
| 6 | 2 | 13 | - 2 | Abdominal pain, vomiting constipation | No mass in one patient, left lower quadrant mass in the other one | Cocoon of whole of small bowel | Lysis in all patients | Well on discharge |
| 20 | 1 | 15 | - 1 | Abdominal pain, distension, vomiting | No mass | Cocoon of whole of small bowel | Resection of caecum and ileocolic anastomosis | Well on discharge |
| 21 | 1 | 15 | - 1 | Symptoms of obstruction | No mass | * | * | * |
| 22 | 1 | 34 | - 1 | Symptoms of obstruction | No mass | * | * | * |
| 4 | 1 | * | 1 - | * | * | * | * | * |
| 23 | 1 | 17 | - 1 | Abdominal pain, vomiting | Mass in right iliac fossa | Cocoon of distal ileum | Lysis | Well on discharge |
| 8 | 4 | 6 - 8 | 3 1 | Abdominal pain, vomiting, constipation, distension | Centrally located abdominal mass in two patients | Cocoon of whole of small bowel in three patients, cocoon of distal ileum, right colon and transverse colon in the other one | Lysis in three patients, resection of the gangrenous bowel and ileocolic anastomosis in the other one | Well on discharge |
| 24 | 1 | 34 | 1 - | Abdominal pain, distension, vomiting | Mass in the umblical region | Cocoon of whole of small bowel | Lysis | Well on discharge |
| 2 | 1 | 12 | - 1 | Abdominal pain, vomiting | Mass in the umblical region and left iliac fossa | Cocoon of whole of small bowel | Lysis | Well on discharge |
| 25 | 5 | 15 - 42 | - 5 | Abdominal pain in all the patients and symptoms of obstruction in two of them | Three patients with abdominal mass | Cocoon of whole of small bowel in two patients and cocoon of distal ileum in three patients | Lysis in four patiens, resection and ileocolic anastomosis in the other one | Well on discharge in four patients (One of them presented two months later with AIDS and died). Other one patient died on the third post-op. day with unknown etiology |
| 26 | 1 | * | * * | * | * | * | * | * |
| 27 | 1 | 38 | 1 - | Symptoms of obstruction, vomiting | Mass in the left half of the abdomen | Cocoon of whole of small bowel which extended to right and left colon | Lysis | Well on discharge |
| 28 | 1 | 65 | 1 - | Abdominal pain, vomiting, constipation | No mass | Cocoon of whole of small | Lysis, resection of distal ileum and ileo-ileal anastomosis | Well on discharge |
| 29 | 1 | 15 | - 1 | * | * | * | * | * |
| 30 | 1 | 16 | 1 - | * | * | * | * | * |
| 31 | 5 | * | 3 2 | * | * | * | * | * |
